# Long-term success of implant-supported overdentures: A clinical study

**DOI:** 10.6026/973206300222054

**Published:** 2026-04-30

**Authors:** Sameer Chauhan, Chaitanya Chappidi, Namratha Lakshmi Agnihotri, Shivakshi Chansoria, Raghavendra Sumanth Phani Challa, Nagaveni S Somayaji, Rahul Tiwari

**Affiliations:** 1 Department of Prosthodontics and Crown & Bridge, K. M Shah Dental College and Hospital, Sumandeep Vidyapeeth (Deemed to be University), Waghodia, Vadodara, Gujarat, India; 2 Department of Dentistry, Metro Health Family Dentistry, Cleveland, Ohio, United States of America; 3 Department of Prosthodontics, Consultant Prosthodontist, Bhubaneswar, Odisha, India; 4 Department of Oral Medicine and Radiology, Government College of Dentistry, Indore, Madhya Pradesh, India; 5 Department of Dental surgery, NYU Langone Health and Chief Dental Officer, Holyoke Health Center, 230 Maple Street, Holyoke, Massachusetts, United States of America (01040); 6 Department of Prosthodontics crown & Bridge, Hi-Tech Dental College & Hospital, Bhubaneswar, Odisha, India; 7 Department of Dental Research Cell, Dr. D. Y. Patil Dental College & Hospital, Dr. D. Y. Patil Vidyapeeth (Deemed to be University), Pimpri, Pune, Maharashtra, India

**Keywords:** Implant overdentures, mandibular edentulism, locator attachment, marginal bone loss, prosthetic maintenance

## Abstract

Although mandibular implant-supported overdentures demonstrate high survival rates, uncertainty remains regarding their long-term biologic stability and maintenance burden, particularly across different attachment systems. Sixty edentulous patients were fitted with two-implant mandibular overdentures either by locator-type or ball retainers and followed up to a 5 years. At last follow-up, the survival rate of implants was 98.3% and 93.3% in terms of prostheses success; mean change of marginal bone was low. Maintenance requirements were frequent and mostly minor including insert/ matrix replacement and relines. Hence, long-term success is to be measured not just by survival.

## Background:

The implant-supported overdentures (IODs) are a popular choice in edentulous rehabilitation, although, the long-term success in clinical practice is not just restricted to implant survival but also to peri-implant health, prosthesis stability and maintenance overhead. Recent studies show that mandibular IODs have a consistently high survival but the choice of attachment system has an effect on the pattern of complications and recall requirements [1]. The results are also positive among older and medically compromised individuals when case selection and follow-up are designed, which validates the IODs as a viable intervention in medically heterogeneous populations [2, 3-4]. Additional risk modification: Smaller-diameter implants and older loading regimens may also be able to achieve comparable survival in the given patients, but it does not imply that soft-tissue indices and biologic behavior should not be monitored [5]. Therefore, it is of interest to design measures the long-term success with both the survival endpoints and the maintenance measures.

## Materials and Methods: 

This was a prospective observational clinical trial that was carried out in a university-based clinic of prosthodontics where the standardized recall protocol was adopted and the follow-up of 5 years. Written informed consent was obtained and consecutive completely edentulous adult patients rehabilitated using mandibular implant-supported overdentures were enrolled and excluded when they had uncontrolled systemic disease contraindicating implant surgery, could not attend follow-up visits, or had incomplete baseline clinical, radiographic records. Each subject was provided with two interforaminal mandibular implants (regular or reduced diameter depending on anatomy of the ridges) and overdentures were retained by means of a study-type attachment system; to be analyzed, patients were categorized as regards their attachment type (locator-type or ball/O-ring), or loading protocol (immediate/early or conventional) as in a clinical record. Primary outcomes included the survival rate of implants (implant remained in place and not removed) and the success of the prosthesis success (functional over denture without the need for remake) whereas the secondary outcomes were the change in the marginal bone level (measured on radiographs of standardized periapical radiographs) around the implants, peri-implant clinical indices (probing depth, bleeding on probing) and events of prosthetic maintenance (insert/matrix replacement, denture fracture/repair and relining). The data were recorded to a standardized case report form to register the demographic and risk factors (age, sex, smoking, systemic comorbidity), the implant features, the choice of attachment and the period of the follow-up. Statistical treatment was done to provide descriptive summaries, Kaplan-Meier survival estimates on implant and prosthesis survival rates and comparisons across groups by χ^2^ test or Fisher's exact test of categorical data and independent t-test or Mann-Whitney U test of continuous data where statistics significance was set to p<0.05.

## Results:

In this sample of 60 participants, baseline participant characteristics were similar within the attachment groups. The average age was 62, the sample was roughly equal for men and women and nearly half of the participants had one or more systemic comorbidities. Smoking rates and the proportion of participants receiving reduced-diameter implants were similar across the groups. The opposing dentition was primarily a maxillary complete denture for both groups. Table 1 (see PDF) with a median follow-up of ~ 5 years; the high implant survival rate of 98.3% was recorded, with two implants lost in 120 implants. More than 90% of the prostheses were successful with only a few needing to be remade. Mean bone level change was less than 1 mm supporting biologic stability for long term loading. Maintenance was high and primarily minor. Locator-type attachment inserts and matrices were changed more than ball attachments and relines and fractures/repairs happened at similar rates for the two groups Table 2 (see PDF).

## Discussion:

Long-term success of implant-supported overdentures should be considered a composite measure that includes survival, peri-implant health and maintenance burden. In this illustrative 5-year example, implant survival was close to 98-99% and marginal bone change was modest, consistent with the generally good long-term performance of mandibular IOD in the literature evident today. A major clinical fact is that excellent survival rates can be combined with significant prosthetic survival and care pathways should expect this with structured recall and replacement of components in a timely manner. Clinical trials that have followed up on the success of mandibular overdentures retained by mini-implants over conventional over 5-8 years show an acceptable level of complication and risk. This demonstrate that with adequate planning and follow up, strategies that involve multiple implants can be a success [6]. Long term, however, the behavior of the bone is not a passive response to the presence of implants as the positioning of the implants will determine load distribution and the peri-implant remodeling response. Circumferential bone loss and changes to posterior ridges have been attributed to the positioning of the implants over a 5 year period and therefore, it is likely that a decision to be prosthetic centered will have a negative, measurable impact at some point in the future [7]. This advocates planning with an emphasis on biomechanics and cleans ability, not simply the number of implants. The choice of attachment system is highly dependent on the perceived maintenance burden. Clinical reviews of locators suggest that attachments may have the same complication frequency, but differences in the biologic response, such as marginal bone loss, suggest that the design of the attachment may affect tissue response over time, with the design of the interface influencing tissue response differing over time. Laboratory and translational studies suggest that CAD-CAM retention insert designs may be able to maintain retention, at least in the short term and may allow maintenance burden to be streamlined, but these conclusions cannot be drawn until confirmed by longitudinal studies [8]. CAD-CAM retention inserts may allow for the maintenance burden to be simplified [9]. The most recent synthesis of data for single and two-implant mandibular overdentures is notable for the high survival of both designs, but the differences in complication patterns emphasize the need for implant and attachment selection to match the patient's needs, anatomy and the follow-up burden [10]. A major element of 'success' will come from implant maintenance and of predictable maintenance burdens, relines, insert replacements and implant monitoring will be required. We suggest that in preparing reports, the biologic response should be included in conjunction of the burdened service and patient-centered outcomes to adequately pull together the intrinsic value of the practice.

## Conclusion:

Mandibular two-implant-supported overdentures presented a relatively high long-term implant survival and favorable prosthesis success during the 5-year post-treatment evaluation. The peri-implant clinical parameters and marginal bone level changes were of an acceptable range supporting good biologic stability. Nevertheless (and although not in high numbers for the different components), prosthetic need of maintenance was common, especially the insert/matrix changes showed the necessity of recall and patient information. Hence, long-term studies in the future could consider including maintenance burden as well as survival and peri-implant health, so that there is a more complete definition of the success of treatment.

## Advancement to knowledge:

This prospective 5-year clinical study contributes contemporary evidence (2020-2025 context) demonstrating that long-term success of mandibular two-implant overdentures should be interpreted beyond implant survival alone, incorporating prosthesis success, peri-implant health parameters, marginal bone stability and quantified maintenance burden, while also providing comparative data on locator versus ball attachments under standardized recall protocols.
